# Cellular Expression of Cyclooxygenase, Aromatase, Adipokines, Inflammation and Cell Proliferation Markers in Breast Cancer Specimen

**DOI:** 10.1371/journal.pone.0138443

**Published:** 2015-10-02

**Authors:** Samar Basu, Kristell Combe, Fabrice Kwiatkowski, Florence Caldefie-Chézet, Frédérique Penault-Llorca, Yves-Jean Bignon, Marie-Paule Vasson

**Affiliations:** 1 Clermont Université, Université d'Auvergne, UMR 1019, Unité de Nutrition Humaine, CRNH-Auvergne, BP 10448, F-63000, Clermont-Ferrand, France; 2 Oxidative Stress and Inflammation, Department of Public Health and Caring Sciences, Faculty of Medicine, Uppsala University, Uppsala, Sweden; 3 Centre Jean Perrin, Unicancer, F-63000, Clermont-Ferrand, France; 4 CHU Clermont-Ferrand, Unité d’exploration nutritionnelle, F-63003, Clermont-Ferrand, France; University of North Carolina School of Medicine, UNITED STATES

## Abstract

Current evidences suggest that expression of Ki67, cyclooxygenase (COX), aromatase, adipokines, prostaglandins, free radicals, β-catenin and α-SMA might be involved in breast cancer pathogenesis. The main objective of this study was to compare expression/localization of these potential compounds in breast cancer tissues with tissues collected adjacent to the tumor using immunohistochemistry and correlated with clinical pathology. The breast cancer specimens were collected from 30 women aged between 49 and 89 years who underwent breast surgery following cancer diagnosis. Expression levels of molecules by different stainings were graded as a score on a scale based upon staining intensity and proportion of positive cells/area or individually. AdipoR1, adiponectin, Ob-R, leptin, COX-1, COX-2, aromatase, PGF_2α_, F_2_-isoprostanes and α-SMA were localised on higher levels in the breast tissues adjacent to the tumor compared to tumor specimens when considering either score or staining area whereas COX-2 and AdipoR2 were found to be higher considering staining intensity and Ki67 on score level in the tumor tissue. There was no significant difference observed on β-catenin either on score nor on staining area and intensity between tissues adjacent to the tumor and tumor tissues. A positive correlation was found between COX-1 and COX-2 in the tumor tissues. In conclusion, these suggest that Ki67, COXs, aromatase, prostaglandin, free radicals, adipokines, β-catenin and α-SMA are involved in breast cancer. These further focus the need of examination of tissues adjacent to tumor, tumor itself and compare them with normal or benign breast tissues for a better understanding of breast cancer pathology and future evaluation of therapeutic benefit.

## Introduction

Breast cancer is a clinically heterogeneous disease [1] linking a variety of complex contributors and interactions that are closely interrelated to indigenous inflammation. There are growing number of evidences suggest that expression of Ki67, cyclooxygenase (COX), aromatase, prostaglandins, free radicals, adipokines, β-catenin and α-SMA might be involved in breast cancer pathogenesis [2–5]. Tumor-associated macrophages harvest a variety of inflammatory mediators that have a vital role in integrity of cellular matrix and proliferation, angiogenesis, invasion and eventual metastasis [6]. It had also been identified that COX-2 is overexpressed in 40% of the invasive human breast cancer, and is related to cell proliferation, metastasis, survival and elevated endogenous prostaglandin levels [7]. Inflammatory mediators such as COX-2 and adipocytokines are also known to involve in the carcinogenesis in a mouse model of mammary cancer and human breast cancer [8,9]. Epidemiological studies suggest that non-steroidal anti-inflammatory drugs (NSAIDs) have some protective effects to human cancer and transgenic COX-2 overexpressed mice induce mammary tumor formation. Further, canonical Wnt/β-catenin signaling seems to play an important role in metastatic breast cancer [10,11].

According to the American Institute for Cancer Research, Washington D.C., advanced obesity is a potential cause of postmenopausal breast cancer which is related to inflammation [12]. Involvement of inflammation, in addition to adipokines and obesity in breast cancer seem to be a convincing theory, since a huge interest has been developed in the current years in studying the function of leptin and adiponectin in mammary tumor growth [13–17]. Both these important proteins/hormones are potential candidates in the process of carcinogenesis, specifically in the obesity-related breast cancer. A significant role is played by the adipose tissue that consists of a blend of mature adipocytes, macrophages and undifferentiated fibroblasts. Therefore, an unstable adipose tissue microenvironment could have an immense impact on breast cancer development [18]. This is probably due to the well-known fact that pro-carcinogenic effect of leptin and anti-carcinogenic effect of adiponectin could be interrelated to the inflammatory response and cell proliferation in the tumor microenvironment [3,15]. Leptin generally acts through its receptor (Ob-R), which is encoded by the Ob gene and activates JAK/STAT (Janus kinase/signal transducer) signaling pathway that increases cell migration and invasion of breast cancer cells. Whereas adiponectin usually initiates cell growth inhibitory effects through its two receptors (AdipoR1 and AdipoR2) via adenosine monophosphate-activated protein kinase (AMPK) pathway. In addition, higher aromatase activity is closely associated to the tumor growth in the breast through estrogen synthesis by the specific stimulation of prostaglandin E_2_ and cAMP signaling [19–23]. Together, breast cancer development is a complex multi-step endogenous regulatory process that includes appearance of various crucial mediators at different progressive stages together with genetic and epigenetic influences of the patient [21,24–26].

On these vital standpoints described above, Ki67, COXs, eicosanoids, aromatase, adipokines, β-catenin and α-SMA are all potential contributors in cell proliferation and breast cancer development. However, no such study has been evaluated these potential mediators expressed *in situ* mutually and their coexistence in tumor microenvironment of human breast cancer. This study describes the direct expression/localization of Ki67, COXs, aromatase, eicosanoids, adipokines and their receptors (Ob-R, AdipoR1 and AdipoR2), β-catenin and α-SMA in breast cancer specimens using immunohistochemistry and correlated with pathological parameters.

## Materials and Methods

### Tissue collection

Breast tissue samples from 30 women aged between 49 and 89 years who underwent surgery in 2009 were obtained from the tumor bank at the Jean-Perrin Anti-Cancer Center, Clermont-Ferrand. The breast tissue samples were obtained from the Jean Perrin Biological Centre, Centre Jean-Perrin, Clermont-Ferrand, France with ethical permission obtained from the French Ministry of Higher Education and Research. The authorization number is BB-0033-00075. Each patient was informed about the use of biological tissue samples for research purposes. If the patient agree on the consent, a non-refusal notice is kept in patient's file. The procedure has been agreed by the Peoples Protection Committee (Nr. AC-2013-1882).

For each patient, two tissue samples were collected: 1) the mammary tumor and, 2) the tissue adjacent to the tumor. Tissue samples were collected during surgery and stored in liquid nitrogen until analysis. The clinical pathological characteristics such as menopausal state, body mass index and tumor histological grade were collected based on pathological reports and medical records.

### Immunofluorescence detection

Tissues were embedded in Optimal Cutting Temperature compound (Tissue-Tek®, Sakura Finetek USA, Torrance, CA) and cutted in sections of 10μm thick using cryostat (Leica, CM1950). After fixation by paraformaldehyde 4% slides were blocked with a solution of PBS 5% donkey serum, 5% Fish skin gelatin, 1% Triton and 0.5% Tween20, and stained by indirect immunofluorescence. First antibodies were diluted in 1% PBS:BSA, 1% Triton, 0.5% Tween20 and incubated overnight at 4°C. The following antibodies rabbit monoclonal anti-COX-2 prediluted (ab21704, Abcam, Cambridge, UK), anti-Ki67 (1:100; AB9260, Millipore, Molsheim, France), anti-β-catenin (1:100, 9582P, Cell Signaling Technology), anti-α-SM22 (1:1000, ab14106, Abcam, Cambridge, UK) were used in the study and are commercially available. Anti-8-iso-prostaglandin-F_2α_ (8-iso-PGF_2α_) and anti-15-keto-13,14-dihydro-prostaglandin-F_2α_ (15-keto-dihydro-PGF_2α_) used at 1:1000 were raised in rabbit as previously described [27,28]. Ethical permission was obtained to raise these antibodies in 1996 with permission from the Ethical Committee, Faculty of Medicine, Uppsala University, Uppsala, Sweden. The authorization number is C 298/96. Antibodies for leptin (1:40, AF398, R&D Systems, Lille, France), anti-Ob-R (1:40, AF389, R&D Systems, Lille, France), anti-AdipoR1 and anti-AdipoR2 (1:100, ab77611 and ab77612, Abcam, Cambridge, UK) and anti-aromatase (1:100, SAB2500110, Sigma, Saint-Louis, USA) were produced in goat. Anti-adiponectin (1:200, ab22554, Abcam, Cambridge, UK) and anti-COX-1 (1:250, ab695, Abcam, Cambridge, UK) were mouse antibodies and are commercially available. For negative controls, sections were incubated without primary antibodies. Tissues were then washed and incubated for one hour at room temperature with 4’,6 diamidino-2-phenylindole (DAPI, 0.5μg/ml) and the second antibody: AlexaFluor 488-conjugated donkey anti-rabbit, or 555-conjugated donkey anti-goat or goat anti-mouse IgG (Invitrogen, Paisley, UK) diluted at 1:1000 in PBS BSA 1% were used in the experiments. After that, sections were mounted in a drop of Mowiol (Calbiochem, France). Part of these stainings were done together with COX-2 and leptine, Ki67 and Adipo R1, β-catenin and AdipoR2, COX-1 and α-SM22 and COX-2 and aromatase. Confocal laser-scanning microscopy was performed with the LeicaTCS SP5 MP confocal and multiphoton microscope system (Leica Inc., Heidelberg, Germany) in collaboration with ICCF (http://www.iccf.fr/). Digital images were processed using Adobe Photoshop CS5.1 and compiled using Adobe Illustrator CS5.1 (Adobe Systems Inc., San Jose, CA). The intensity of the stainings were visually appreciated such as-, +/-, +, +/++, ++, ++/+++, +++ and converted to number (- = 0, +/- or + or +/++ = 1 and ++ or ++/+++ or +++ = 2). The average number of stained positive cells (Ki67) or positive area (all other stainings) per microscopic field (magnification 20X) was quantified using the particle analyzer from ImageJ software (National Institutes of Health, Bethesda, MD, USA). Images were subjected to the threshold function and then the number of particles in the image or the area was counted. Particles less than 10 pixels were excluded from the study.

### Interpretation of immunofluorescence staining

Expression levels of the molecules by different stainings were graded as a score on a scale of 0 to 2 based on staining intensity and proportion of positive cells or positive area. The staining was scored as 0 if no cells were reactive, 1 if staining was weakly positive with <2/3 of positive cells or strongly positive with <1/3 positive cells and 2 if staining was weakly positive with >2/3 positive cells or strongly positive with >1/3 positive cells. The final scores were classified as negative (score 0) or positive (1 or 2). For Ki67 staining, data were expressed as the ratio Ki67^+^ nucleus number on total nucleus number. For β-catenin staining, data were expressed as the ratio number of tissues samples stained either in nucleus, or in cytoplasm, or both on total number of tissues samples analyzed.

### Statistical analysis

The data were expressed as the mean ± SEM. Differences between tissues adjacent to the tumor and tumor tissues groups were assessed using Student paired T test. Pearson test was used to check correlation between two biomarkers. Spearman test, test H, H Kruskall and Wallis were used for β-catenin expression and correlation study with clinical parameters. Similarly, Pearson test, test H Kruskall and Wallis, Mann and Whitney U test were used for Ki67, COX-2 expression and correlation study with clinical parameters. Overall survival of patients and COX-2 was estimated by Chi^2^ test.

## Results

### Clinical characteristics of the patients

Clinical characteristics of the patients are shown in Table 1. The mean age of the patients was 65 years (range, 49–89). Menopausal state of the patients was as follows: 4 patients were of premenopausal state (13%), 24 patients were of postmenopausal (80%) state and it is unknown for 2 subjects. The mean BMI of the patients was 26.2 kg/m^2^ (range 20.8–37.6 kg/m^2^). A total of 13 patients were overweighted (BMI >25 kg/m^2^, according to the World Health Organisation) and 15 patients were of normal weight (BMI 18.5–24.9 kg/m^2^). 27 (90%) patients were of ductal carcinoma, 1 patient was of lobular carcinoma and 2 patients of dual character. Positive lymph node involvement were in 13 cases (43.3%). Histologic grades of the tumor were as follows: grade 1 in 5 cases (16.7%); grade 1–2 in 2 cases (6.7%), grade 2 in 13 cases (43.3%) and grade 3 in 10 cases (33.3%). Estrogen receptor (ER) positive was in 29 cases (97%) and progesterone receptor (PR) positive was in 25 cases (83%). No overexpression of HER-2 was seen in 28 cases (93%).

**Table 1:** Clinical characteristics of patients (n = 30).

Variables		Number	(range, %)
Age (year)		65	(49–89)
BMI (kg/m^2^)		26.2	(20.8–37.6)
18.5 to 25		15	(50)
25 to 30		8	(26.7)
30 to 35		3	(10)
35 to 40		2	(6.7)
Unknown		2	(6.7)
Menopausal state	Premenauposal	4	(13.3)
	Postmenauposal	24	(80)
	Unknown	2	(6.7)
Type	Ductal invasive	27	(90)
	Lobular invasive	1	(3.3)
	Dual invasive	2	(6.7)
Lymph node involvement	Negative	17	(56.7)
	Positive	13	(43.3)
Histologic grade	Grade 1	5	(16.7)
	Grade 1–2	2	(6.7)
	Grade 2	13	(43.3)
	Grade 3	10	(33.3)
ER expression	Negative	1	(3.3)
	Positive	29	(96.7)
PR expression	Negative	5	(16.7)
	Positive	25	(83.3)
HER-2 expression	No overexpression	28	(93.3)
	Overexpression	2	(6.7)

BMI = Body Mass Index; ER = Estrogen Receptor; PR = Progesterone Receptor; HER = Human

epidermal growth factor receptor

### Immunofluorescence staining data

**Fig 1:**
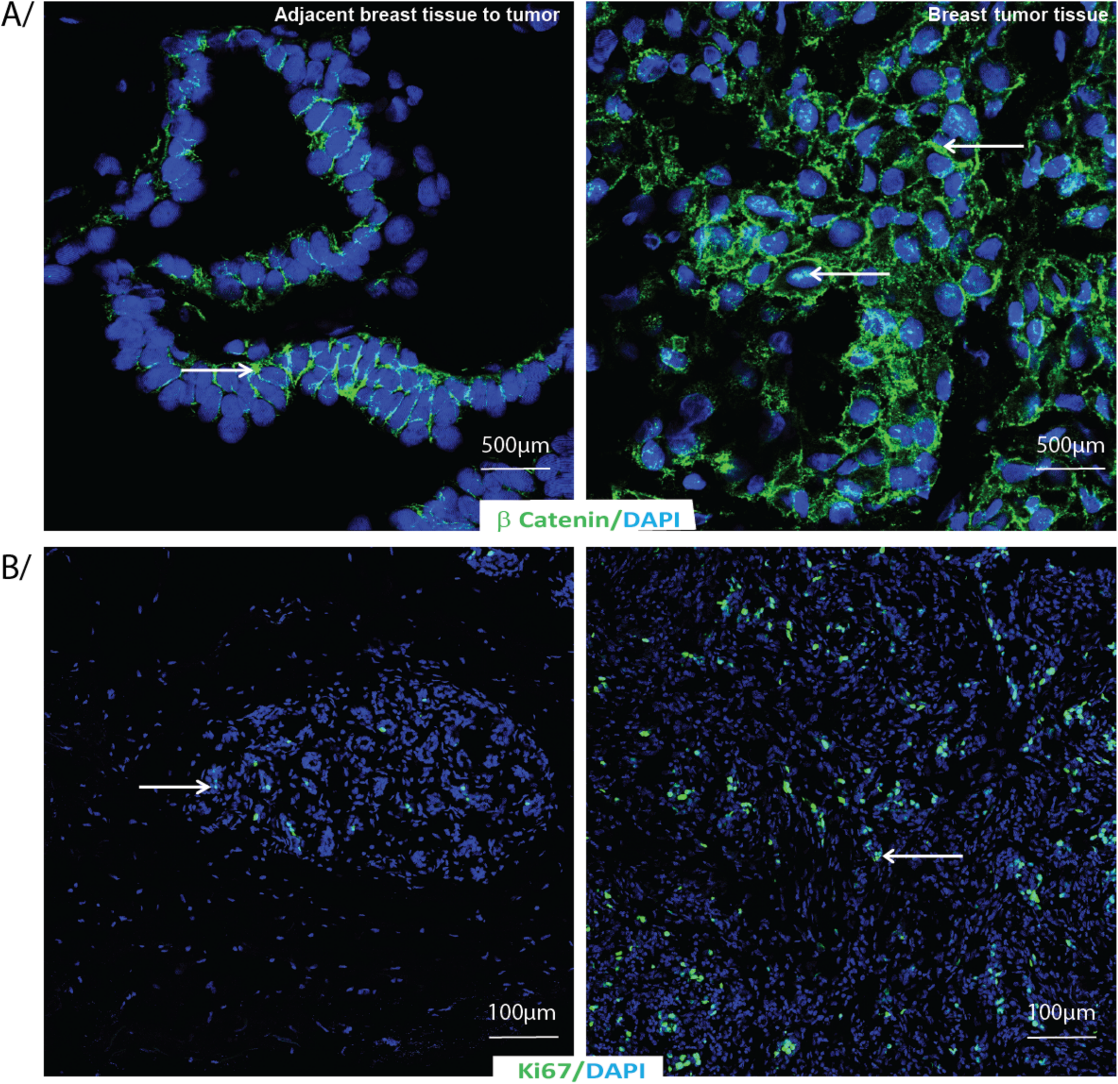
β-Catenin and Ki67 immunostaining in adjacent breast tissue to tumor or breast tumor tissue. Adjacent breast tissue to tumor or breast tumor tissue labeled by indirect immunofluorescence for A/ β-catenin and B/ Ki67 (green) with DAPI as nuclear counterstain (blue).

Significant higher level of cell proliferation marker Ki67 was found in the nucleus of tumor tissue (6.90±4.74) than in the tissue adjacent to the tumor (1.18±1.19) (p = 0.0001) (Table 2). Similarly we observed more β-catenin staining in the nucleus of the tumor tissue than in the nucleus of the tissue adjacent to the tumor (2/3 cells with cytoplasmic staining in the healthy tissue adjacent to the tumor and 1/3 cells with cytoplasmic staining in the tumor tissue). Fig 1 shows adjacent breast tissue to tumor (left panel) and breast tumor tissues (right panel) labeled by indirect immunofluorescence for A) β-catenin and B) Ki67 with DAPI as nuclear counterstain.

**Table 2:** Comparison of proliferation markers (Ki67 and β-Catenin) in breast tissues adjacent to the tumor and tumor tissues of breast cancer patients based on immunohistochemistry staining.

Biomarkers	Breast tumor tissue (n = 30)				Adjacent breast tissue to tumor (n = 30)				Paired T test p<0.05
Ratio (Ki67^+^ nucleus / total nucleus)	6.90±4.74				1.18±1.19				0.0001
β-Catenin	C	C + N		N	C	C + N		N	0.02
	n = 10	n = 18		n = 2	n = 19	n = 9		n = 2	
		C (% area*)	N (% area)			C (% area)	N (% area)		
		69	31			51	49		

C: cytoplasma; N = nucleus; n = number of samples

* % area stained.

**Fig 2:**
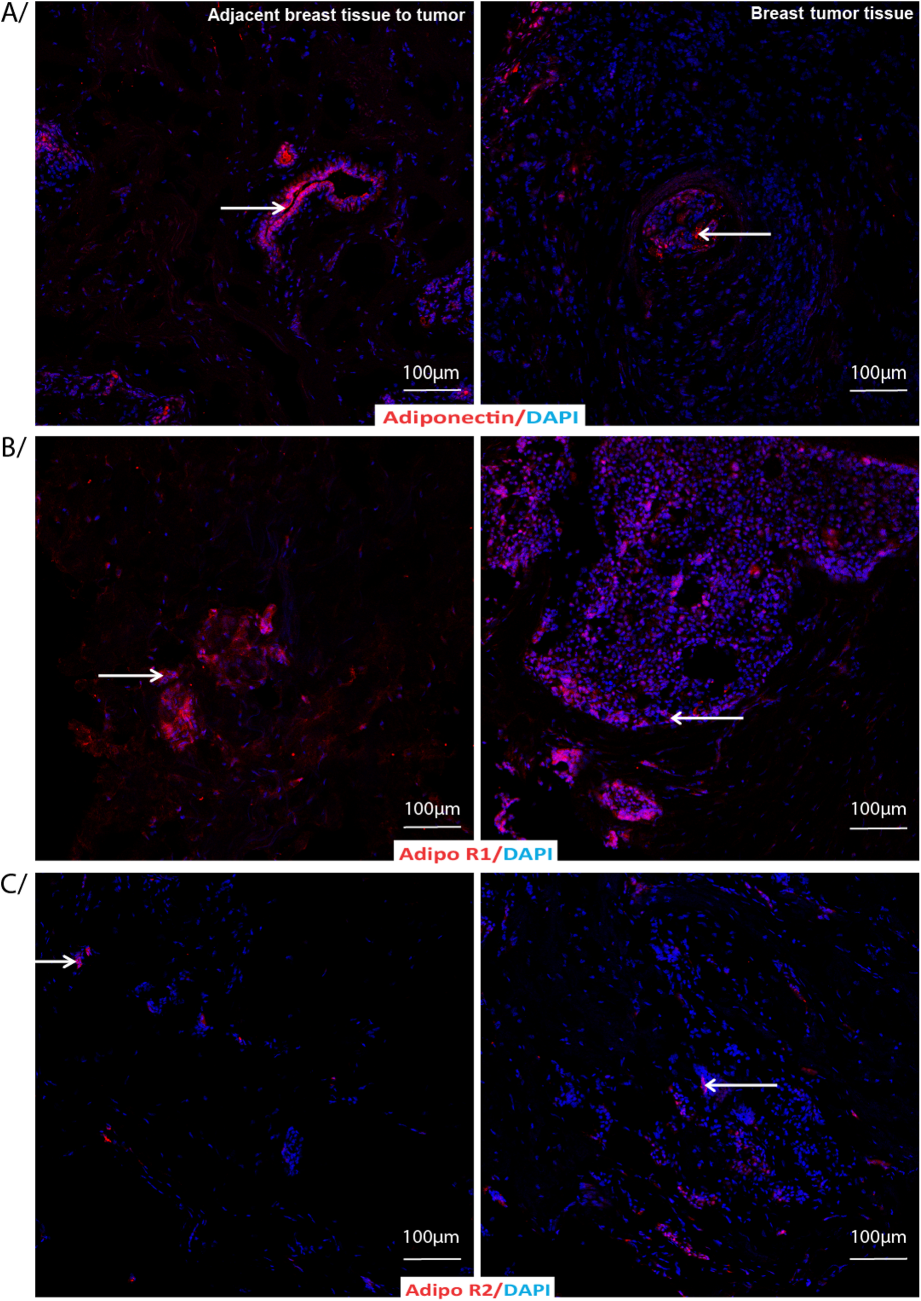
Adiponectin and Adiponectin receptors immunostaining in adjacent breast tissue to tumor or breast tumor tissue. Adjacent breast tissue to tumor or breast tumor tissue labeled by indirect immunofluorescence for A/ Adiponectin, and B/ Adiponectin receptor 1 and C/ Adiponectin receptor 2 (red) with DAPI as nuclear counterstain (blue).

**Fig 3:**
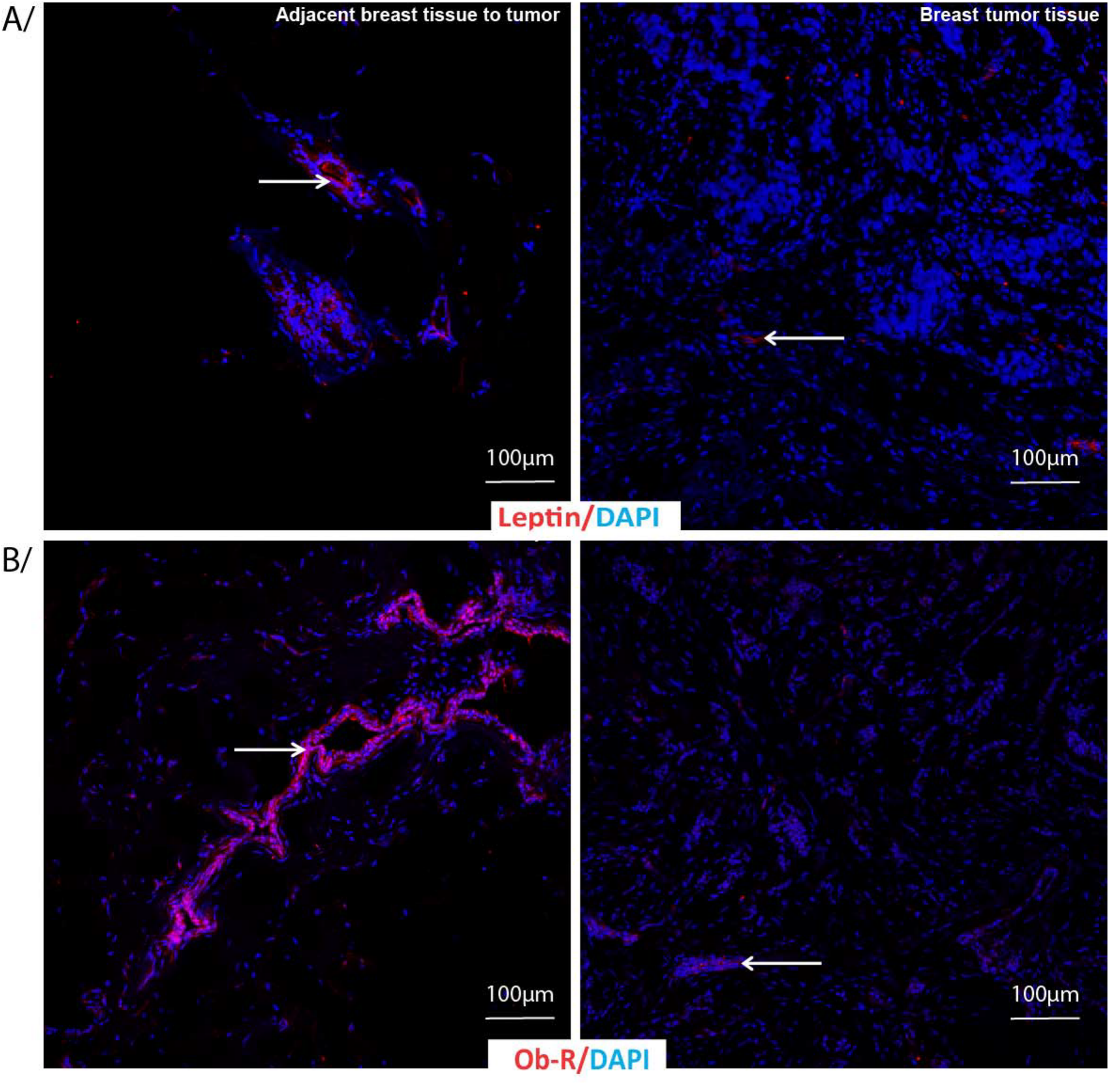
Leptin and Leptin receptor immunostaining in adjacent breast tissue to tumor or breast tumor tissue. Adjacent breast tissue to tumor or breast tumor tissue labeled by indirect immunofluorescence for A/ Leptin and B/ Leptin receptor Ob-R (red) with DAPI as nuclear counterstain (blue).

Table 3 shows a comparison between tissues adjacent to the tumor and tumor tissues collected from breast cancer patients on different parameters based on the score calculated from the percent of total stained area and intensity of immunostaining. A representative figure shows adjacent breast tissue to tumor (left panel) and breast tumor tissues (right panel) labeled by indirect immunofluorescence for A) Adiponectin and B) AdipoR1 and AdipoR2 with DAPI as nuclear counterstain (Fig 2). AdipoR1 was significantly lower in the tumor tissue than the tissues adjacent to the tumor (p = 0.027). Ob-R, the leptin receptor levels were significantly lower in the tumor tissue than the tissues adjacent to the tumor (p = 0.01). In addition, both leptin and adiponectin levels were also significantly lower in the tumor tissue compared to the tissues adjacent to the tumor (leptin, p = 0.027; adiponectin, p = 0.0023). Ratio between adiponectin and leptin levels were indifferent comparing either of the tissues (0.89±0.96 in the tumor tissue and 1.03±095 in the tissue adjacent to the tumor, p = ns). Fig 3 shows adjacent breast tissue to tumor (left panel) and breast tumor tissues (right panel) labeled by indirect immunofluorescence for A) Leptin and B) Leptin receptor Ob-R with DAPI as nuclear counterstain.

**Table 3:** Comparison of adipokines and their receptors (AdipoR1, AdipoR2 and Ob-R), COXs, F_2_-isoprostanes, prostaglandin F_2α_, α-SMA and aromatase in breast tissues adjacent to the tumor and tumor tissues of breast cancer patients based on a score calculated from the staining intensity and percent of area immunostained tissues.

	Breast tumor tissues		Adjacent breast tissues to tumor		
Biomarkers	n	*Score*	n	*Score*	Paired T test p<0.05
AdipoR1	27	0.93±0.27	25	1.24±0.36	0.027
AdipoR2	30	0.83±0.28	29	0.83±0.34	ns
Adiponectin	29	0.90±0.19	25	1.15±0.46	0.0023
Ob-R	30	1.00±0.00	28	1.21±1.34	0.01
Leptin	30	1.07±0.12	27	1.26±0.38	0.027
COX-1	28	1.18±0.29	26	1.38±0.47	ns
COX-2	27	0.70±0.42	24	0.95±0.09	0.016
F_2_-isoprostanes	29	0.48±0.50	25	0.64±0.51	0.026
PGF_2α_	29	0.55±0.49	26	0.73±0.39	0.05
α-SMA	30	0.79±0.55	27	1.19±0.42	0.027
Aromatase	26	1.04±0.22	26	0.96±0.07	ns

Results are the mean ± SEM; ns = not significant; Adipo R1/R2 = adiponectin receptor; Ob-R = leptin receptor; COX = cyclooxygenase; PGF_2α_ = prostaglandin F_2α_; α-SMA = α-Smooth Muscle Actin

**Fig 4:**
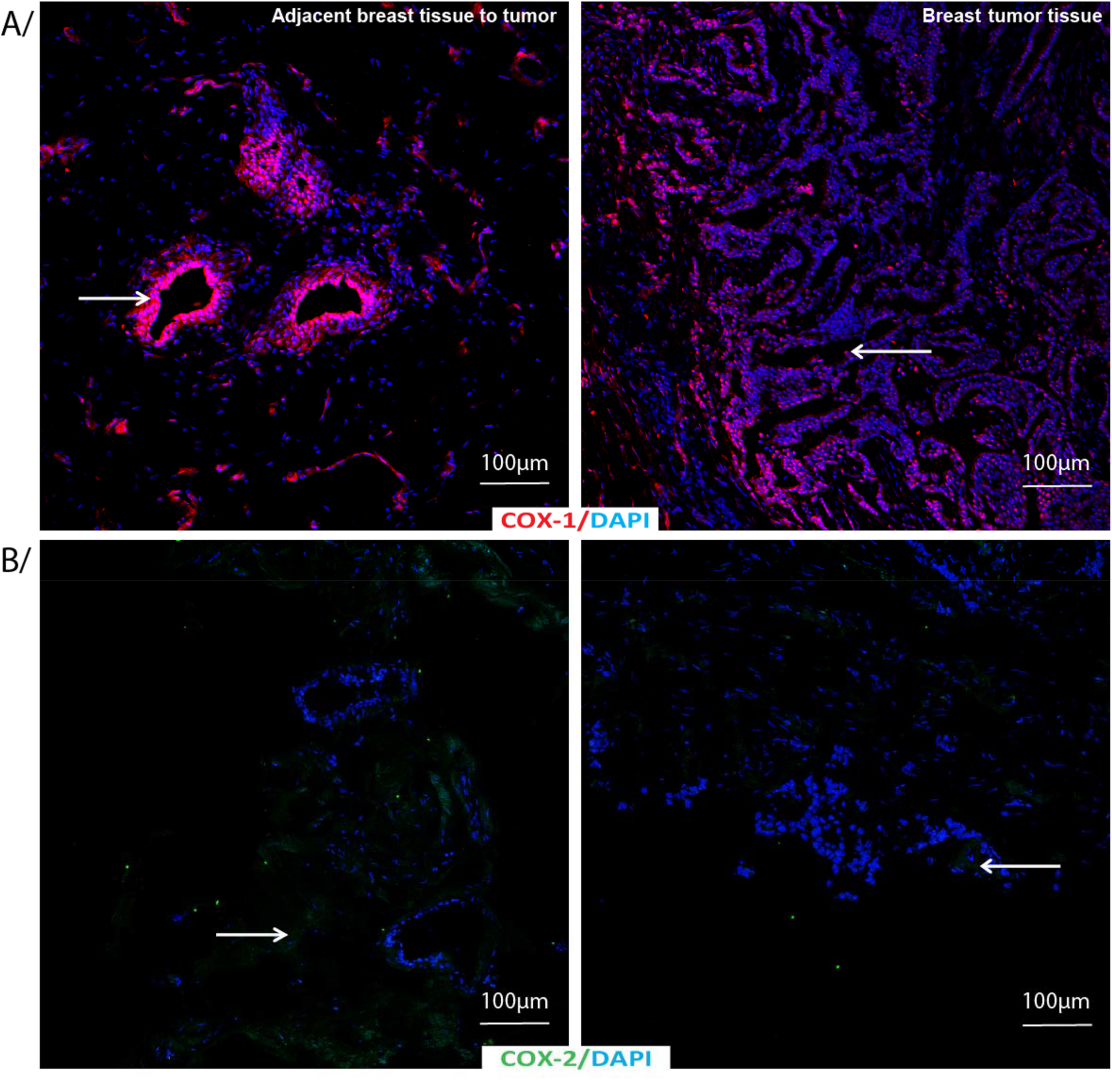
COX-1 and COX-2 immunostaining in adjacent breast tissue to tumor or breast tumor tissue. Adjacent breast tissue to tumor or breast tumor tissue labeled by indirect immunofluorescence for A/ COX-1 (red) and B/ COX-2 (green) with DAPI as nuclear counterstain (blue).

COX-1, being the constitutive enzyme that keeps the homeostasis and prostaglandin biosynthesis was not different in the tissues adjacent to the tumor compared to the tumor tissue. COX-2, F_2_-isoprostanes and PGF_2α_ were lower in the tumor tissue than tissues adjacent to the tumor (COX-2, p = 0.016; 8-iso-PGF_2α_, p = 0.026; PGF_2α_, p = 0.05). Fig 4 shows adjacent breast tissue to tumor (left panel) and breast tumor tissues (right panel) labeled by indirect immunofluorescence for A) COX-1 and B) COX-2 with DAPI as nuclear counterstain. α-SMA was higher in the tissues adjacent to the tumor than tumor tissues (p = 0.027) and aromatase was indifferent comparing either of the tissues.

Tables 4 and 5 show a comparison between tissues adjacent to the tumor and tumor tissues collected from breast cancer patients on different parameters based on the percent of total stained area and intensity of immunostaining, respectively. AdipoR1 was significantly higher in the tissues adjacent to the tumor (p = 0.0031) compared to the tumor tissues when considering percent of area stained whereas indifferent levels were found when compared on the staining intensity levels. AdipoR2 showed an opposite spectrum than AdipoR1 on intensity levels than percent of total stained area. Adiponectin values were significantly higher (p = 0.022) and leptin was lower (p = 0.00005) on intensity levels in the tissues adjacent to the tumor compared to the tumor tissues but both adiponectin and leptin values were higher (adiponectin, p = 0.0009; leptin, p = 0.0000001) in the tissues adjacent to the tumor compared to the tumor tissues on the percent of stained area. Ob-R (p = 0.0000001) values was higher on the percent of area stained in the tissues adjacent to the tumor than tumor tissues whereas no such difference was observed when counting staining intensity.

**Table 4:** Comparison of adipokines and their receptors (AdipoR1, AdipoR2 and Ob-R), COXs, aromatase, F_2_-isoprostanes, prostaglandin F_2α_, α-SMA and aromatase in breast tissues adjacent to the tumor and tumor tissues of breast cancer patients on the basis of percent of staining area.

	Breast tumor tissues		Adjacent breast tissues to tumor		
Biomarkers	n	% area stained	N	% area stained	Paired T test p<0.05
AdipoR1	27	11.71±10.67	25	24.97±13.87	0.0031
AdipoR2	30	7.15±6.34	29	8.97±7.57	ns
Adiponectin	29	4.07±3.64	25	10.47±7.85	0.0009
Ob-R	30	9.02±6.51	28	23.20±11.37	<0.0000001
Leptin	30	9.90±7.46	27	23.74±19.45	<0.0000001
COX-1	28	17.00±10.62	26	35.23±20.35	0.0015
COX-2	27	1.09±1.13	24	3.41±3.87	0.0096
F_2_-isoprostanes	29	1.21±1.42	25	6.42±6.73	0.0048
PGF_2α_	29	2.19±2.89	26	6.68±5.16	0.012
α-SMA	30	12.40±15.24	27	23.81±16.79	ns
Aromatase	26	8.22±8.97	26	15.94±9.93	0.0067

Results are the mean ± SEM; ns: not significant. AU = Arbitrary Unit; Adipo R1/R2 = adiponectin receptor; Ob-R = leptin receptor; COX = cyclooxygenase; PGF_2α_ = prostaglandin F_2α_; α-SMA = α-Smooth Muscle Actin

**Table 5:** Comparison of adipokines and their receptors (AdipoR1, AdipoR2 and Ob-R), COXs, F_2_-isoprostanes and prostaglandin F_2α_, α-SMA and aromatase in the breast tissues adjacent to the tumor and tumor tissues of breast cancer patients on the basis of staining intensity.

	Breast Tumor tissues		Adjacent breast Tissue to tumor		
Biomarkers	n	Intensity (AU)	n	Intensity (AU)	Paired T test p<0.05
AdipoR1	27	1.30±0.63	25	1.37±0.47	ns
AdipoR2	30	1.27±0.64	29	0.86±0.36	0.022
Adiponectin	29	1.14±0.42	25	1.50±0.55	0.0023
Ob-R	30	1.57±0.49	28	1.46±0.50	ns
Leptin	30	1.80±0.32	27	1.33±0.44	0.00005
COX-1	28	1.86±0.24	26	1.69±0.43	ns
COX-2	27	0.63±0.51	24	0.33±0.44	0.009
F_2_-isoprostanes	29	0.59±0.61	25	0.76±0.61	0.025
PGF_2α_	29	0.72±0.65	26	1.04±1.59	0.037
α-SMA	30	1.17±0.86	27	1.67±0.49	0.02
Aromatase	26	1.50±0.58	26	1.32±0.48	ns

Results are the mean ± SEM; ns: not significant. AU = Arbitrary Unit; Adipo R1/R2 = adiponectin receptor; Ob-R = leptin receptor; COX = cyclooxygenase; PGF_2α_ = prostaglandin F_2α_; α-SMA = α-Smooth Muscle Actin

**Fig 5:**
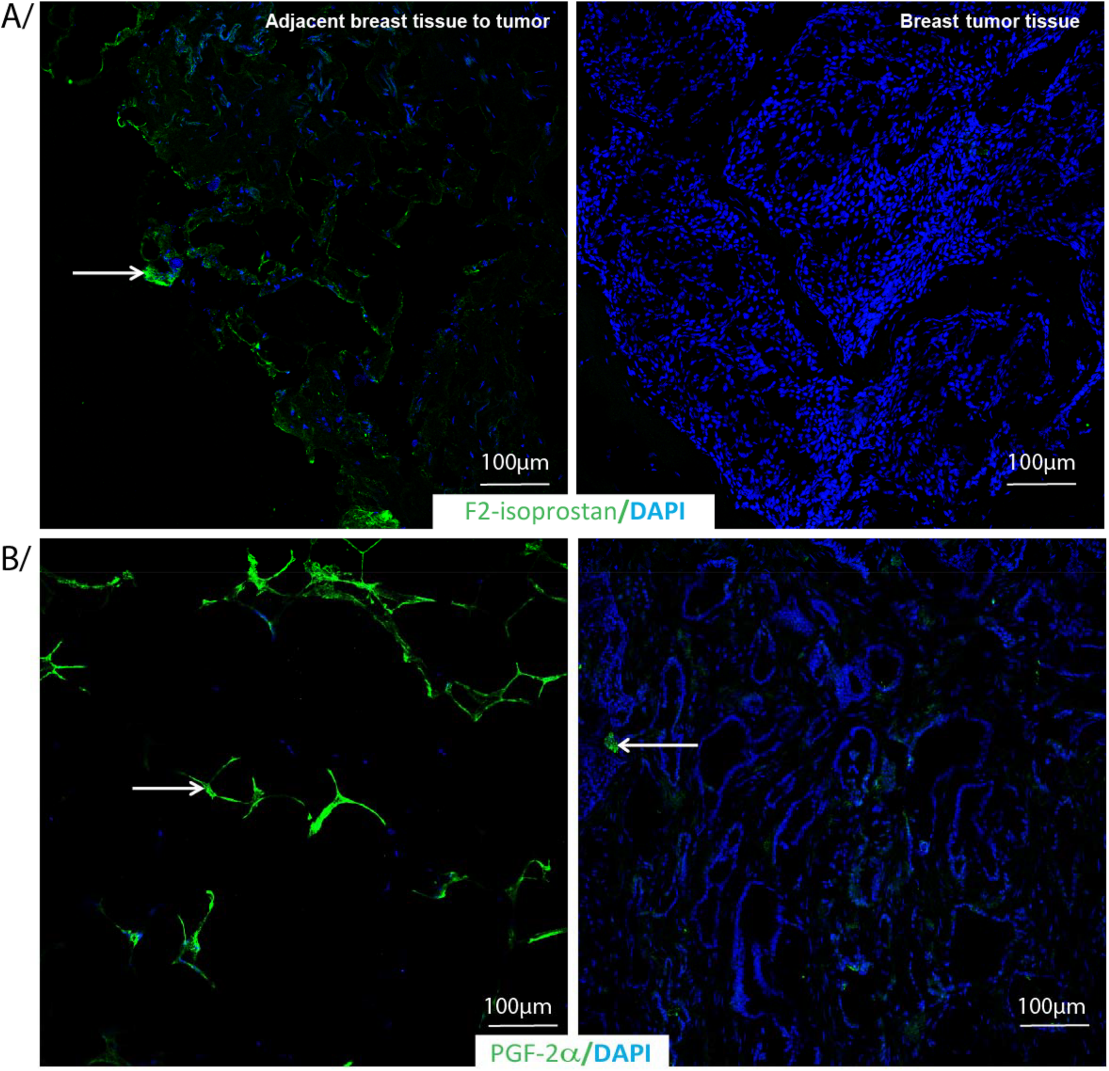
F_2_-isoprostane and PGF_2α_ immunostaning in adjacent breast tissue to tumor or breast tumor tissue. Adjacent breast tissue to tumor or breast tumor tissue labeled by indirect immunofluorescence for A/ F_2_-isoprostane and B/ PGF_2α_ (green) with DAPI as nuclear counterstain (blue).

COX-1 values were lower in the tumor tissues than tissues adjacent to the tumor (p = 0.0015) on the percent of stained area. There was less COX-2 in the tumor than in the tissues adjacent to the tumor (with a large SEM) (p = 0.0096) considering the percent area stained as seen for the score above. However when considering the intensity, COX-2 values are significantly lower (p = 0.009) for tissues adjacent to the tumor compared to tumor tissues. F_2_-isoprostanes and PGF_2α_ metabolite levels were higher both on the percent of area stained (F_2_-isoprostanes, p = 0.0048; PGF_2α_, p = 0.012) and intensity levels in the tissues adjacent to the tumor than tumor tissues (F_2_-isoprostanes, p = 0.025; PGF_2α_, p = 0.037). Fig 5 shows adjacent breast tissue to tumor (left panel) and breast tumor tissues (right panel) labeled by indirect immunofluorescence for A) F_2_-isoprostanes and B) PGF_2α_ with DAPI as nuclear counterstain.

Using multivariate survival analysis considering the whole population, the overall survival is found to be 87.7% at 5 years. When considering two groups selected by COX-2 median value, we observe no significant difference for overall survival for tissue adjacent to the tumor, whereas the underexpression of COX-2 is associated with lower but no significant overall survival with a follow up of 4.7 years (p = 0.38) for tumor tissue. Thus with regard to the small number of patients, this study cannot conclude on the survival rate.

**Fig 6:**
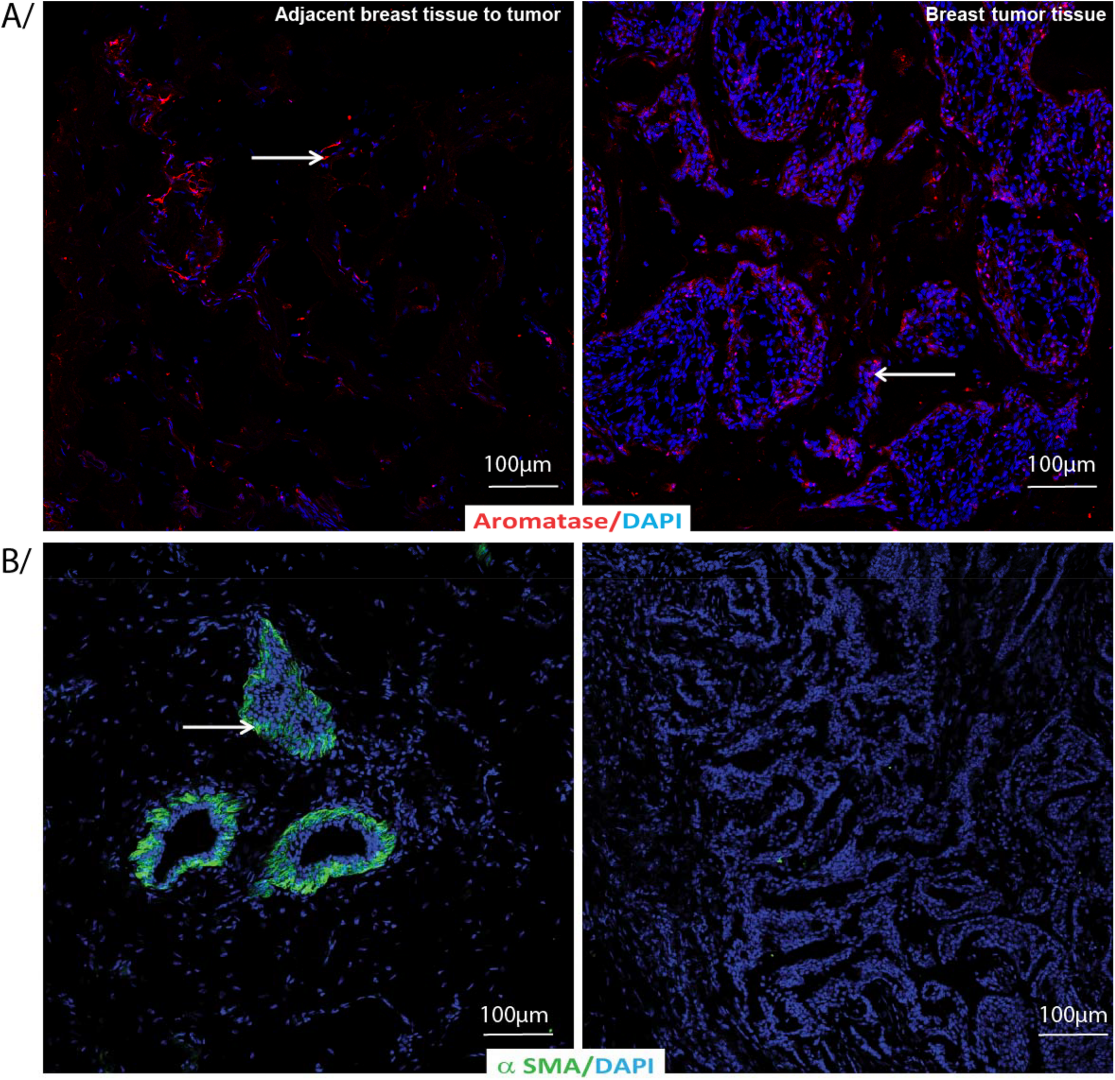
Aromatase and α-SMA immunostaning in adjacent breast tissue to tumor or breast tumor tissue. Adjacent breast tissue to tumor or breast tumor tissue labeled by indirect immunofluorescence for A/ Aromatase (red) and B/ α-SMA (green) with DAPI as nuclear counterstain (blue).

α-SMA levels were higher (p = 0.02) in the tissues adjacent to the tumor than the tumor tissues on intensity level but not on staining area. Aromatase (p = 0.0067) values was higher on the percent of area stained in the tissues adjacent to the tumor than tumor tissues whereas no such difference was observed when counting staining intensity. Fig 6 shows adjacent breast tissue to tumor (left panel) and breast tumor tissues (right panel) labeled by indirect immunofluorescence for A) Aromatase and B) α-SMA with DAPI as nuclear counterstain.

Since some of the women in this study are postmenopausal we have analysed the aromatase score values selecting the two groups of post- and premenopausal women. For postmenopausal the aromatase score is: breast tumor tissues (n = 22), 1.05 +/- 0.49; and adjacent breast tissue to tumor (n = 22), 1.00 +/- 0.00; When performed paired T test no significance is found (p = 0.79). For premenopausal women the aromatase score is: breast tumor tissues (n = 3), 1.00 +/- 0.00; and adjacent breast tissue to tumor (n = 4), 0.75 +/- 0.43; When performed Mann and Whitney test no significance has been obtained (p = 0.50). Thus, the menopausal status does not seem to confound the analysis.

### Correlations between cyclooxygenases and adipokines, aromatase and prostaglandins

Correlations between COX-1 and AdipoR1, AdipoR2, adiponectin, leptin, aromatase, PGF_2α_ metabolite were shown in Table 6. No correlation was found between these parameters. Correlations between COX-2 and AdipoR1, AdipoR2, adiponectin, leptin, aromatase, PGF_2α_ metabolite are shown in Table 7. However, a positive correlation was found between COX-1 and COX-2 in the tumor tissues.

**Table 6:** Correlation between staining score values of COX-1 and adipokines and their receptors, aromatase and PGF_2α_ in adjacent breast tissue and tumor tissues as performed by Pearson test.

	COX-1									
Biomarkers	Breast tumor tissues (Mean±SEM: 1.18±0.29, n = 28)					Adjacent breast tissue to tumor (Mean±SEM: 1.38±0.47, n = 26)				
	Mean±SEM	n	r	ddl	p	Mean±SEM	n	r	ddl	p
AdipoR1	0.93±0.27	27	-0.152	23	0.470	1.24±0.36	25	-0.258	19	0.260
AdipoR2	0.83±0.28	30	0.217	26	0.270	0.83±0.34	29	0.062	23	0.770
Adiponectin	0.90±0.19	29	-0.135	25	0.510	1.15±0.46	25	0.048	22	0.820
Leptin	1.07±0.12	30	-0.900	26	0.650	1.26±0.38	27	-0.098	22	0.650
Aromatase	1.04±0.22	26	0.000	22	1.000	0.96±0.07	26	0.167	23	0.430
PGF_2α_	0.55±0.49	29	0.780	25	0.700	0.73±0.39	26	0.065	22	0.760

COX-1 = cyclooxygenase-1; Adipo R1/R2 = adiponectin receptor; PGF_2α_ = prostaglandin F_2α_

**Table 7:** Correlation between staining score values of COX-2 and adipokines and their receptors, aromatase, COX-1 and PGF_2α_ in healthy and tumor tissues as performed by Pearson test.

	COX-2									
Biomarkers	Breast tumor tissues (Mean±SEM: 0.70±0.42, n = 27)					Adjacent breast tissue to tumor (Mean±SEM: 0.53±0.50, n = 24)				
	Mean±SEM	N	r	ddl	p	Mean±SEM	N	r	ddl	p
AdipoR1	0.93±0.27	27	0.033	25	0.860	1.24±0.36	25	-0.185	23	0.380
AdipoR2	0.83±0.28	30	0.000	28	1.000	0.83±0.34	29	0.128	27	0.510
Adiponectin	0.90±0.19	29	0.174	27	0.370	1.15±0.46	25	-0.051	24	0.800
Leptin	1.07±0.12	30	-0.055	28	0.770	1.26±0.38	27	-0.239	25	0.230
Aromatase	1.04±0.22	26	-0.106	24	0.610	0.96±0.07	26	0.143	26	0.470
COX-1	1.18±0.29	28	-0.389	26	0.039	1.38±0.47	26	-0.013	24	0.950
PGF_2α_	0.55±0.49	29	-0.133	27	0.500	0.73±0.39	26	0.217	24	0.290

COX = cyclooxygenase; Adipo R1/R2 = adiponectin receptor; PGF_2α_ = prostaglandin F_2α_

### Correlation between β-catenin, Ki67, Her2/neu and clinical pathology

Correlation between β-catenin and age is significant regardless the type of tissue (tumor or healthy: globally, p = 0.0005). All other correlations are not significant considering the tumor volume (= tumor diameter), the degree of differentiation (histologic grade), the lymph node involvement and the tumor-node-met staging. As expected, correlation with Ki67 and histologic grade (or SBR) is found to be significant (p = 0.0045). Correlations with other clinical pathology parameters are not significant considering the age, the tumor volume (= tumor diameter), the lymph node involvement and the tumor-node-met staging. Correlation between Her2/neu and clinical pathology parameters is not possible to perform due to only two tumors overexpressed this parameter (Table 1).

## Discussions

In this study we have observed a higher level of cell proliferation marker, Ki67 in the tumor tissue which is in accordance with earlier studies [2, 29] and also correlates with histologic grade. Ki67 is a nuclear protein that is encoded with MK 167 gene which is associated with and prerequisite for cell proliferation. This is a prominent marker to elucidate the growth of a certain cell population. Ki67 protein is present in all active phases of the cell cycle process, and is usually absent from resting cells. The fraction of Ki67-positive tumor cells is generally associated with the clinical outcome of cancer, such as breast and prostate cancer [29,30]. In a coherent way, the nuclear or cytoplasmic staining of the β-catenin, a protein of the Wnt pathway, also shows a proliferation more important in the tumor cells than in the cells of the tissue adjacent to the tumor. Indeed, the nuclear localization of β-catenin shows an activation of the Wnt pathway, a major way of cell proliferation.

This study demonstrate that adiponectin, AdipoR1, leptin, Ob-R, COX-1, COX-2, aromatase, F_2_-isoprostanes, PGF_2α_ metabolite and α-SMA are localised on higher levels in the breast tissues adjacent to the tumor compared to tumor specimens either on a score calculated from both the staining area and intensity or individually on the staining area or intensity of staining. More specifically, when evaluating score alone AdipoR1, adiponectin, Ob-R, leptin, COX-2, F_2_-isoprostanes, PGF_2α_ and α-SMA were also higher in the tissues adjacent to the tumor compared to the tumor tissues. Further, Ki67 was found in higher levels in the tumor tissues. The results also show that AdipoR1, adiponectin, Ob-R, leptin, aromatase, COX-1, COX-2, F_2_-isoprostanes and PGF_2α_ were higher in the tissues adjacent to the tumor compared to the tumor tissues based on percent of staining area whereas AdipoR2, leptin and COX-2 were higher in the tumor tissues compared to the breast tissues adjacent to the tumor based on the percent of staining intensity. Breast tissues adjacent to the tumor showed higher levels of adiponectin, F_2_-isoprostanes, PGF_2α_ and α-SMA compared to the tumor tissues based on the percent of staining intensity. Collectively, these results suggest that expression of these potential pathophysiological mediators (adipocytokines and their receptors, COXs, aromatase, Ki67, and α-SMA, F_2_-isoprostanes and PGF_2α_) are confined either in the adjacent tissues to the breast tumor or in the tumor itself. This disparate identification of the compounds through different detections procedures might indicate a differential availability of these compounds in the microenvironment of both in the tumor and the tissues adjacent to the tumor. This manifestation may possibly have an influence on the appearance and disappearance of systemic levels of these compounds and also on biological effects at various stages of the disease in patients. These distinctive appearances of these compounds in the cells most possibly have further impact on the growth of the tumor and subsequently intensifying their stimulus to the neighboring tissues.

AdipoR1, adiponectin, Ob-R and leptin were higher on score and area of staining in the tissues adjacent to the tumor than the tumor tissue. However, higher intensity of staining was seen for leptin in the tumor than in the tissues adjacent to the tumor as shown in the earlier studies [9,31,32]. When determining intensity alone adiponectin, PGF_2α_, F_2_-isoprostanes levels were higher in the tissues adjacent to the tumor than the tumor tissue whereas AdipoR2, leptin, and COX-2 levels were higher in the tumor tissues. Higher levels of α-SMA were also seen in the tissues adjacent to the tumor compared to the tumor both on the score and intensity of staining.

COX-2, COX-mediated product PGF_2α_ and F_2_-isoprostanes, a free radical mediated product of arachidonic acid on score values and aromatase, COX-1, COX-2, PGF_2α_, F_2_-isoprostanes on percent of staining area levels were also higher in the tissues adjacent to the tumor than the tumor tissue. Similar higher levels on intensity were also found for PGF_2α_ and F_2_-isoprostanes. Some eicosanoids specifically promote further development and growth of breast tumor indirectly by inducing aromatase, particularly in the estrogen positive breast cancer [33], and nearly all cases presented here (29 out of 30) belong to this group. Earlier it has shown that higher levels of COX-2 in the tumor tissues than in normal mammary gland tissue [5,33,34] as it is seen in the current study when we consider intensity of staining. These differential appearances perhaps are due to the fundamental molecular alterations occurring that are reflected by histologically characterized so called normal appearing adjacent tissues. The question then arises how “normal healthy” the tissues are adjacent to the breast tumor. This issue on the selection of tissues for scientific judgement has earlier been highlighted in other studies [35,36]. Tissues adjacent to the primary breast cancer tissues could be not of same character when using normal or benign breast tissue as a comparison which we have not determined in our study. However, other study has indicated that adjacent breast specimens from triple-negative breast cancer, which were all positive for CD44^+^CD49fCD13372^+^ stem cell staining, also exhibited high tumorigenic signature patterns [36]. Similarly, it has also been described that immunohistochemical expression of COX-2, IL-10 and Ki67 was higher in tumor stromal areas than tumor cell areas [37]. These observations certainly highlight that adjacent normal tissue to the tumor tissues have already acquired a number of transcriptional changes comparable to the tumor tissues. Further, these studies together emphasis that microenvironment surrounding tumor epithelium is of vital importance to scrutiny and plays a crucial role in breast cancer progression [18]. An appropriate normal breast tissue as a baseline comparison with adjacent tumor tissue and the tumor tissue itself will essentially give a more precise indication of the disease dissemination to the neighboring cells. A positive correlation was found between COX-1 and COX-2 in the tumor tissues in this study which is expected since both these enzymes are closely-related subtypes. Both COXs are well connected to inflammatory environments and are responsible for subsequent prostaglandin formation [38,39]. Together, this study presents the presence of Ki67, AdipoR1, AdipoR2, adiponectin, Ob-R, leptin, aromatase, COX-1, COX-2, PGF_2α_, F_2_-isoprostanes and α-SMA are localised on higher levels in the breast tissues adjacent to the tumor or in the tumor itself which may have pathological consequences in the breast cancer.

This study has some limitations regarding the small number of patient samples inclusion. Further, we conducted immunohistochemical analysis only on the breast that undergone surgery in breast cancer diagnosed patients but not compared with healthy breast tissues. Thus, we had any baseline normal or benign breast tissue sample for comparison with the tumor tissues mainly due to ethical reasons of access of healthy breast tissue samples. In addition, there remains always a risk of misclassification of the single section with the histopathological pattern of the specimen that may sometime overlook the localisation. However, the sections were always been carefully collected and the results were cautiously judged by an experienced pathologist to determine the authenticity and characteristics of the sample. Despite these limitations, our study has several strengths, such as we have evaluated and presented the results in the cancer microenvironment through immunohiostochemical positive staining cells/area or intensity of staining individually and also through a careful judgement of the score calculated from both the intensity and the staining area. In addition, we have determined several potential variants of parameters in the same samples that are currently regarded as important mediators and contributors in breast cancer pathology. Further, the majority of the patients were at postmenopausal stage diagnosed with estrogen and prosgesterone receptor positive and having ductal carcinoma, those are the important criteria’s that coincide with many of the selected variables and parameters we have studied.

In conclusion, this immunohistochemical study shows that AdipoR1, adiponectin, Ob-R, leptin, aromatase, COX-1, COX-2, PGF_2α_, F_2_-isoprostanes and α-SMA are expressed and localised on higher levels in the breast tissues adjacent to the tumor compared to tumor specimens when considering either score or staining area whereas AdipoR2, leptin and COX-2 were also found on staining intensity and Ki67 on score level in the tumor tissue. Further these findings heighten the need of investigation of adjacent tumor microenvironment together with tumor and normal or benign breast tissues to understand the multifaceted existence and interactions of numerous mediators in breast cancer pathology and future evaluation of therapeutic benefit.
